# Establishment of a retinoic acid-resistant human acute promyelocytic leukaemia (APL) model in human granulocyte-macrophage colony-stimulating factor (hGM-CSF) transgenic severe combined immunodeficiency (SCID) mice.

**DOI:** 10.1038/bjc.1998.596

**Published:** 1998-10

**Authors:** Y. Fukuchi, M. Kizaki, K. Kinjo, N. Awaya, A. Muto, M. Ito, Y. Kawai, A. Umezawa, J. Hata, Y. Ueyama, Y. Ikeda

**Affiliations:** Hu-Mouse Project, Eighth Laboratory, Kanagawa Academy of Science and Technology, Japan.

## Abstract

**Images:**


British Journal of Cancer (1998) 78(7), 878–884
© 1998 Cancer Research Campaign

### Establishment of a retinoic acid-resistant human acute promyelocytic leukaemia (APL) model in human granulocyte–macrophage colony-stimulating factor (hGM-CSF) transgenic severe combined immunodeficiency (SCID) mice

Y Fukuchi[1,2], M Kizaki[3], K Kinjo[3], N Awaya[3], A Muto[3], M Ito[1,2], Y Kawai[4], A Umezawa[5], J Hata[5], Y Ueyama[1,2,6] and Y Ikeda[3]

[1]Hu-Mouse Project, Eighth Laboratory, Kanagawa Academy of Science and Technology, Kanagawa, Japan; [2]Central Institute for Experimental Animals, Kanagawa, Japan; [3]Departments of Internal Medicine, [4]Clinical Laboratories and [5]Pathology, Division of Haematology, Keio University School of Medicine, Tokyo, Japan; [6]Department of Pathology, School of Medicine, Tokai University, Kanagawa, Japan

**Summary** To understand the mechanisms and identify novel approaches to overcoming retinoic acid (RA) resistance in acute promyelocytic leukaemia (APL), we established the first human RA-resistant APL model in severe combined immunodeficiency (SCID) mice. UF-1 cells, an RA-resistant APL cell line established in our laboratory, were transplanted into human granulocyte–macrophage colony-stimulating factor (GM-CSF)-producing SCID (hGMTg SCID) mice and inoculated cells formed subcutaneous tumours in all hGMTg SCID mice, but not in the non-transgenic control SCID mice. Single-cell suspensions (UF-1/GMTg SCID cells) were similar in morphological, immunological, cytogenetic and molecular genetic features to parental UF-1 cells. All-*trans* RA did not change the morphological features of cells or their expression of CD11b. RA did not alter the growth curve of cells as determined by MTT assay, suggesting that UF-1/GMTg SCID cells are resistant to RA. These results demonstrate that this is the first RA-resistant APL animal model that may be useful for investigating the biology of this myeloid leukaemia in vivo, as well as for evaluating novel therapeutic approaches including patients with RA-resistant APL.

**Keywords:** acute promyelocytic leukaemia; severe combined immunodeficiency mice; human granulocyte–macrophage colony-stimulating factor; retinoic acid; drug resistance

All-*trans* retinoic acid (RA) can induce terminal differentiation of the leukaemic cells, resulting in complete remission in most patients with acute promyelocytic leukaemia (APL) (Huang et al, 1988; Kanamaru et al, 1995; Warrell et al, 1991). However, the majority of patients became RA resistant with continuous treatment with RA (Warrell et al, 1993). Mechanisms and strategies to overcome RA resistance in APL are still unclear, and it is a serious clinical problem for differentiation-inducing therapy. Recently, we established a novel APL cell line (UF-1) with RA-resistant features that will be a useful model for studies on the block of differentiation of the leukaemic cells (Kizaki et al, 1996*a*). However, studies based on the analysis of cell lines in vitro may not reflect in vivo conditions. Thus, a suitable in vivo model for human APL is critical for investigating mechanisms of RA resistance and to develop novel therapeutic drugs for patients.

Severe combined immunodeficient (SCID) mice have been used as a model for studying the biology of human disease. Unlike lymphoid leukaemic cells, human myeloid leukaemic cells have been difficult to propagate in SCID mice (Uckun et al, 1996). It has been shown that cytokines, and fetal bone and thymus, increase the reconstitution capacity of the human haematopoietic system in SCID mice (Lapidod et al, 1994). To address these problems, we have established human granulocyte–macrophage colony-stimulating factor (GM-CSF)-producing transgenic SCID (hGMTg SCID) mice (Miyakawa et al, 1996). In this report, we demonstrate and characterize the first human RA-resistant APL model using UF-1 cells and hGMTg SCID mice.

*Received 9 December 1997*
*Revised 10 March 1998*
*Accepted 18 March 1998*

*Correspondence to:* M Kizaki, Division of Haematology, Keio University School of Medicine, 35 Shinanomachi, Shinjuku-ku, Tokyo 160-8582, Japan

### MATEIALS AND METHODS

### Cells and chemicals

The RA-resistant APL cell line (UF-1) was established in our laboratory (Kizaki et al, 1996*a*). RA-sensitive HL-60 and NB4 cells (the latter a gift from Dr M Lanotte, Hôpital St. Louis, Paris, France) (Lanotte et al, 1991) were maintained in RPMI-1640 medium (Gibco-BRL, Gaitherburg, MD, USA) containing 15% fetal bovine serum (FBS; Hyclone Laboratories, Logan, UT, USA), 100 U $ml^{-1}$ penicillin and 100 μg $ml^{-1}$ streptomycin in a humidified atmosphere with 5% carbon dioxide. All-*trans* RA was purchased from Sigma Chemical Co. (St. Louis, MO, USA) and dissolved in 100% ethanol to a stock concentration of 1 mM, stored at –20°C and protected from light.

### Transgenic (Tg) SCID mice producing human GM-CSF

Human GM-CSF transgenic (hGMTg) SCID mice used in this study were newly produced as described previously (Miyakawa et al, 1996). Briefly, equal numbers of pCDSRαhGM-CSF and

**Figure 1** Histopathology and morphology of subcutaneous tumour in the UF-1-injected SCID mouse. (**A** and **B**) Section of subcutaneous tumour in the hGMTg SCID mouse with diffuse infiltration of leukaemic cells. Tissue was fixed in 10% formalin and embedded in paraffin, and then stained with haematoxylin and eosin [original magnification × 40 (**A**) and × 400 (**B**)]. (**C** and **D**) Morphology of parental UF-1 (**C**) and UF-1/GMTg SCID cells (**D**). Single-cell suspensions (UF-1/GMTg SCID cells) were collected and cytospin slides were prepared and stained with Giemsa [original magnification × 1000 (**C** and **D**)]

pCDSRαhIL-3 plasmids (provided by Dr Y Takebe, National Institute of Infectious Diseases, Tokyo, Japan) (Takebe et al, 1988) linearized with *Apa*LI (Takara Shuzo, Tokyo, Japan) were mixed and microinjected into prenuclear stage embryos obtained by crossing BDF1 females with C.B-17-*scid* or C57B6/J (B6J)-*scid* males. A Tg mouse producing hGM-CSF in the sera born of two founder mice, which carried both pCDSRαhGM-CSF and pCDSRαhIL-3 in their genomes, was crossed with B6J-*scid* to obtain hGM-CSF producing *scid/scid* offspring. The mice were maintained by back-crossing with B6J-*scid* under specific pathogen-free conditions in our laboratory. Serum levels of human GM-CSF were measured using an enzyme-linked immunosorbent assay (ELISA) kit (R&D Systems, Minneapolis, MN, USA) in triplicate according to the manufacturer's instruction.

### Inoculation of UF-1 cells into hGMTg SCID mice

UF-1 cells ($1 \times 10^7$ cells) were inoculated either intraperitoneally (i.p.) or subcutaneously (s.c.) into hGMTg SCID and control B6J SCID mice. Mice were pretreated with 3 Gy of total-body irradiation, a sublethal dose that may enhance acceptance of xenografts (Miyakawa et al, 1996). Leukaemic cell growth was assessed by daily measurements of the dimension of subcutaneous nodules. When the animals showed severe wasting, they were not observed further and the day of sacrifice was recorded to estimate lifespan according to the UKCCCR guidelines (Workman et al, 1988). Surviving mice were sacrificed at the end of the experiment and sectioned for microscopic examination of the tumour.

### Analysis of leukaemic cells and tissue infiltration

### *Morphology and histopathology*

A leukaemic tumour nodule was removed from SCID mice, cut into small pieces with a scalpel and then gently ground in a nylon cell strainer within a tissue culture dish containing RPMI-1640 medium. Single-cell suspensions (UF-1/GMTg SCID cells) were collected and morphology was evaluated from cytospin slide preparations with Giemsa stain. To estimate the infiltration of the leukaemic cells in different organs, tissue sections from mice were fixed in 10% formalin and paraffin embedded, and then stained with haematoxylin and eosin.

### *Cytogenetic studies*

Chromosomes of UF-1/GMTg SCID cells were analysed by standard Giemsa banding techniques as described previously (Kamada et al, 1981).

© Cancer Research Campaign 1998

**Table 1** Engraftment of UF-1 cells in hGMTg SCID mice

| Mice | Sex | Age (weeks) | Serum levels of hGM-CSF ($pg\ ml^{-1}$) | Route of inoculation | Engraftment (days after inoculation) | Findings |
|---|---|---|---|---|---|---|
| hGMTg SCID | | | | | | |
| 1 | M | 13 | 6440 | s.c. | + (27) | Subcutaneous tumour · |
| 2 | M | 13 | >10 000 | i.p. | + (52) | Subcutaneous tumour, ascites. Intraabdominal tumour |
| 3 | F | 10.5 | 6640 | s.c. | + (20) | Subcutaneous tumour |
| 4 | F | 10.5 | 6760 | i.p. | + (59) | Subcutaneous tumour, abscess |
| B6J SCID | | | | | | |
| 1 | M | 10.5 | – | s.c. | – | |
| 2 | M | 10.5 | – | i.p. | – | |
| 3 | F | 10.5 | – | s.c. | + (83) | Subcutaneous tumour |
| 4 | F | 10.5 | – | i.p. | – | (tumour/total body weight 1.6%) |

Twenty-four hours after 3 Gy of TBI, SCID mice were injected s.c. or i.p. with $1 \times 10^7$ UF-1 cells.

### *Surface marker analysis*

Cell-surface antigens were detected by immunofluoresence staining with monoclonal antibodies including CD3, CD4, CD5, CD7, CD8, CD10, CD11b, CD13, CD14, CD19, CD20, HLA-DR, CD33, CD34, CD38 and CD41 (Becton Dickinson, Mountain View, CA, USA). The cells were analysed by flow cytometry (FACScan, Becton Dickinson) and the data represent the mean of triplicate experiments.

### *RT-PCR assay for PML/RARα fusion transcript*

RT-PCR assay for PML/RARα was carried out with both parental UF-1 and UF-1/GMTg SCID cells, as well as control HL-60 cells as previously described (Kizaki et al, 1996*a*).

### *FISH analysis*

To confirm the cytogenetic findings, fluorescence in situ hybridization (FISH) was performed on slides from the same culture as cytogenetics with specific DNA probes for RARα and PML (Hiorns et al, 1994).

### *Assays for cellular proliferation and differentiation*

Parental UF-1 and UF-1/GMTg SCID cells, as well as NB4 cells, were cultured with all-*trans* RA ($10^{-10}$–$10^{-6}$ M) for 4 days. Cellular proliferation was measured using a non-radioactive cell proliferation assay system (MTT assay; Boehringer Mannheim, Indianapolis, IN, USA) according to the manufacturer's protocol. For analysis of cellular differentiation, cells were examined by morphology using Giemsa staining and expression of cell-surface CD11b. Cells were incubated for 60 min with human AB serum (Sigma) to block Fc receptors and then stained with phycoerythrin (PE)-conjugated mouse anti-human CD11b antibody (Becton Dickinson) (Kizaki et al, 1996*b*).

### RESULTS

### Inoculation of UF-1 cells into hGMTg SCID mice

RA-resistant human APL cells (UF-1) ($1 \times 10^7$) were injected either intraperitoneally (i.p.) or subcutaneously (s.c.) into four hGMTg SCID mice and four control B6J SCID mice. Endogenous serum human GM-CSF levels were detected in all hGMTg SCID mice (6640–10 000 $pg\ ml^{-1}$) but not in any non-transgenic B6J SCID mice (Table 1). UF-1 cells formed tumours at the injected site as subcutaneous tumours in 4 out of 4 hGMTg SCID mice between days 20 and 59 (Table 1). Subcutaneous tumour, intra-abdominal tumour and ascites were developed in only one mouse with i.p. injection (Table 1). All of the tumours were composed of leukaemic cells; however, no obvious infiltration was observed in the major organs in both hGMTg SCID and control B6J mice (Figure 1A and B, and data not shown). Only 1 out of 4 control mice developed a small subcutaneous tumour; this was found at autopsy (Table 1).

### Morphology of UF-1/GMTg SCID cells

Single-cell suspensions from tumours were collected and cultured in RPMI-1640 medium. Cells proliferated without any haematopoietic growth factors and were designated as UF-1/GMTg SCID cells. UF-1/GMTg SCID cells were similar in morphology to parental UF-1 cells. UF-1/GMTg SCID cells and parental UF-1

**Table 2** Reactivity of parental UF-1 cells and UF-1/GMTg SCID cells with monoclonal antibodies

| Cells | Monoclonal antibodies (% of positive cells) | | | | | | | | | | | | | | | |
|---|---|---|---|---|---|---|---|---|---|---|---|---|---|---|---|---|
| | CD3 | CD4 | CD5 | CD7 | CD8 | CD10 | CD11b | CD13 | CD14 | CD19 | CD20 | HLA-DR | CD33 | CD34 | CD38 | CD41 |
| Parental UF-1 | <1 | <1 | 2 | 91 | 5 | 2 | 1 | 38 | 22 | <1 | <1 | 1 | 91 | <1 | 65 | 1 |
| UF-1/GMTg SCID | 1 | 1 | <1 | 85 | <1 | 1 | 8 | 35 | 1 | <1 | 1 | 1 | 8 | 1 | 44 | 1 |

 © Cancer Research Campaign 1998

**Figure 2** Karyotype of UF-1/GMTg SCID cells, showing 46, XX, add(1)(q44), add(6)(q12), add(7)(q36) and t(15;17)(q22;q11–12)

cells showed large and often lobulated nuclei with a few nucleoli, and contained large azurophilic granules that were compatible with hypergranulocytic promyelocytes (Figure 1C and D).

### Surface marker analysis

Phenotypic analysis using various monoclonal antibodies in parental UF-1 and UF-1/GMTg SCID cells is summarized in Table 2. Leukaemic cells displayed the same phenotype as parental cells. Both types of cells were positive for CD7, CD13 and CD38. Interestingly, parental UF-1 cells were 91% positive for CD33, whereas UF-1/GMTg SCID cells were only 8% positive (Table 2).

### Cytogenetic and FISH studies

Cytogenetic analysis of G-trypsin-banded karyotypes was performed on 20 metaphases. Both UF-1/GMTg SCID cells and parental UF-1 cells showed t(15;17) (q22;q11–12) and additional abnormalities of add(1)(q44), add(6)(q12) and add(7)(q36) (Figure 2). To confirm the cytogenetic studies, we performed FISH analysis using metaphase chromosomes from UF-1/GMTg SCID cells. PML/RARα fusion signals were detected in all samples (Figure 3).

### RT-PCR analysis of PML/RAR-α fusion transcripts

We also examined the expression of PML/RAR-α chimeric transcript in UF-1/GMTg SCID cells by using RT-PCR. The PML/RAR-α transcript was detected in both parental UF-1 and UF-1/GMTg SCID cells, but not in HL-60 cells (negative control). These results were confirmed by subsequent Southern blotting of the PCR products (Figure 4).

### Effects of all-*trans* RA on proliferation and differentiation of NB4, parental UF-1 and UF-1/GMTg SCID cells

RA-sensitive NB4 cells, parental UF-1 and UF-1/GMTg SCID cells were incubated with all-*trans* RA ($10^{-10}$ to $10^{-6}$ M) for 4 days. All-*trans* RA inhibited cellular proliferation of NB4 cells in a dose-dependent manner (Figure 5A). By contrast, the absorbance of MTT was changed more gradually in parental UF-1 and UF-1/GMTg SCID cells, suggesting that all-*trans* RA did not affect cell growth at $10^{-10}$ to $10^{-8}$ M RA. However, cell proliferation decreased by 30% and 40%, respectively, after parental UF-1 and UF-1/GMTg SCID cells were exposed to higher concentrations of all-*trans* RA ($10^{-6}$ M) (Figure 5A).

Induction of differentiation of these cell lines into mature granulocytes by all-trans RA was assessed by morphology and expression of CD11b using FACS analysis (Figure 5B). NB4 cells were differentiated towards mature granulocytes by RA, whereas all-*trans* RA did not cause morphological differentiation of parental UF-1 and UF-1/GMTg SCID cells towards either mature granulocytes or monocytes (data not shown). CD11b expression in NB4 cells was increased by RA in a dose-dependent manner. In contrast, all-*trans* RA did not alter CD11b expression in UF-1/GMTg SCID cells except at $10^{-6}$ M. These results were similar to those obtained in parental UF-1 cells, suggesting that both parental UF-1 and UF-1/GMTg SCID cells were resistant to induction of cellular differentiation by all-*trans* RA.

### DISCUSSION

APL is characterized by the t(15;17) translocation, which fuses the PML gene on chromosome 15 to the RAR-α gene on chromosome 17, and this PML/RAR-α fusion transcript may be involved in the leukaemogenesis of APL (Warrell et al, 1993). All-*trans* RA is now being used in the treatment of APL as differentiation-inducing therapy (Huang et al, 1988; Warrell et al, 1991; Kanamaru et al, 1995). Although a high proportion of patients with APL achieve complete remission with all-*trans* RA, most patients will develop early clinical relapse and eventual resistance to retinoids (Warrell et al, 1993). To date, most approaches have not been successful in overcoming RA resistance in patients; thus, a suitable animal model of APL is important.

SCID mice provide a model system to study the biology of human leukaemias and explore the feasibility and efficacy of novel therapeutic approaches to leukaemia. Many studies have

© Cancer Research Campaign 1998

**Figure 3** FISH on a representative metaphase from UF-1/GMTg SCID cells. Arrows show the PML gene (red) and the RAR-α gene (green). and the arrowhead indicates the specific signal of the fusion gene

**Figure 4** RT-PCR analysis of PML/RARα fusion transcript. Total RNA was extracted from HL-60 cells (lane 1). parental UF-1 cells (lane 2) and UF-1/GMTg SCID cells (lane 3). RT-PCR products were electrophoresed. transferred and hybridized with PML/RAR-α- and β-actin-specific probes. The 324-bp product indicates the presence of PML/RAR-α fusion transcript. and subsequent hybridization with β-actin probe is performed as a control for RT-PCR products in each lane been done to establish a SCID model of human leukaemias. including acute myelocytic leukaemia (AML). However. unexplained graft failures have been observed. and inoculation of myeloid leukaemic cells into SCID mice has generally been less successful than lymphoid leukaemic cell inoculation (Lord et al. 1991: Sawyers et al. 1992: Namikawa et al. 1993). Therefore. several investigations have attempted to improve engraftment of human myeloid leukaemic cells in SCID mice using various haematopoietic growth factors and human fetal tissues (Terpstra et al. 1995). Recently. Lapidot et al (1994) have reported that primary leukaemic cells injected into sublethally irradiated SCID mice that were treated with cytokines (PIXY321 and SCF) resulted in highly reproducible engraftment in mice. To resolve these problems. we have established transgenic mice producing human GM-CSF with homozygous *scid* gene (hGMTg SCID mice) (Miyakawa et al. 1996). We have already shown that functional hGM-CSF αβ receptor-transfected Ba/F3 cells formed tumours and invaded organs in this hGMTg SCID model. suggesting that these mice would be a useful model for human myeloid leukaemias. In the present study. we created a human RA-resistant APL SCID mouse model using this system.

The growth pattern of subcutaneously inoculated human primary myeloid leukaemic cells in SCID mice has correlated with clinical outcomes (Yan et al. 1996). APL cells have strikingly low proliferation potential in vitro: therefore. leukaemic cells from patients with APL are more difficult to transplant into SCID mice than other types of myeloid leukaemia (Cesano et al. 1992: Yan et al. 1996). We have previously established and characterized a novel APL cell line (UF-1) with RA-resistant features (Kizaki et al. 1996*a*). This cell line had an enhanced proliferation in the pres-

 © Cancer Research Campaign 1998

**Figure 5** Comparison of the proliferation and differentiation-inducing activities of all-*trans* RA on RA-sensitive NB4 cells, RA-resistant parental UF-1 and UF-1/GMTg SCID cells. (**A**) MTT assay. Cells were cultured in the presence of all-*trans* RA ($10^{-10}$ to $10^{-6}$ M) for 4 days and MTT incorporation was measured. Results are expressed as a per cent of control absorbance and the mean of three experiments: the s.d. was 10% of the mean. (**B**) Expression of CD11b on NB4, parental UF-1 and UF-1/SCID cells. Cells were treated for 4 days with all-*trans* RA and then analysed by FACS. Data represent the means of triplicate experiments and the s.d. was within 10% of the mean ence of GM-CSF, IL-3 and SCF, but not G-CSF, M-CSF, IL-6, and TGF-β in vitro (Kizaki et al, 1996*a*). Thus, UF-1 cells were transplanted into our hGMTg SCID mice, and we could establish a human APL mouse model, and single-cell suspensions (UF-1/GMTg SCID cells) were obtained from tumours. To date, there has been only one APL mouse model using RA-sensitive NB4 cells and SCID mice (Zhang et al, 1996). NB4 cells ($1 \times 10^6$ cells) were transplanted into the peritoneum of SCID mice and then appeared as NB4 ascites cells (A-NB4), which differentiated into mature granulocytes by all-*trans* RA (Zhang et al, 1996). In contrast to A-NB4 cells, all-*trans* RA did not change the morphological features and CD11b expression or growth rate of UF-1/GMTg SCID cells, indicating that these cells are resistant to RA. Thus, these mice are the first human RA-resistant APL animal model.

UF-1/GMTg SCID cells were positive for CD7, CD13 and CD38 and negative for CD34. These results are similar to parental UF-1 cells (Kizaki et al, 1996*a*). Interestingly, CD33 and CD14 were expressed in parental UF-1 cells but not in UF-1/GMTg SCID cells. The reason for this finding is unclear. Because CD14 is preferentially expressed in monocyte-like cells, a certain change in cell phenotype might be occurring. In addition, perhaps the CD33-negative leukaemic cells had a growth advantage in vivo during establishment and development of this APL mice model. It has been reported that leukaemic cell proliferation and high levels of blast colony-forming units (AML-CFU) were observed in CD34-positive cells, and CD34-negative cells were poorly engrafted into SCID mice (Lapidod et al, 1994), suggesting that expression of CD34, but not CD33, is important for engraftment. Consistent with this report, we could not reproducibly inoculate UF-1 cells into control B6J SCID mice. In marked contrast, UF-1 cells were successfully engrafted in human GM-CSF, producing hGMTg SCID mice. We also have successfully transplanted a variety of leukaemic cell lines, including NB4 (promyelocytic leukaemia; Lanotte et al, 1991), UT-7 (megakaryocytic leukaemia; Komatsu et al, 1991) and TF-1 (erythroblastic leukaemia; Kitamura et al, 1989), into these transgenic SCID mice (data not shown). In particular, UT-7 and TF-1 cells are leukaemic cell lines that require GM-CSF and IL-3 for growth and survival. These results suggest that this mouse system is more adaptive to myeloid leukaemic cells and may be a useful in vivo model of human myeloid leukaemia. The leukaemic cells spreading to the haematopoietic tissues, including bone marrow and peripheral blood, would be an ideal model for human leukaemia. Therefore, further studies and additional treatment of hGMTg SCID mice will be needed to develop an ideal animal model.

In summary, using hGMTg SCID mice, we established a human RA-resistant APL mouse model. In addition to intra-abdominal and ascites manifestations of APL, we successfully transplanted UF-1 cells into hGMTg SCID mice as subcutaneous tumours. Recent clinical and in vitro studies in China have shown that arsenic trioxide is an effective and safe drug in the treatment of APL patients refractory to all-*trans* RA (Chen et al, 1996). However, no animal studies on arsenic trioxide exist to determine the lethal dose and detailed pharmacokinetics in vivo. Therefore, this RA-resistant APL model will be useful for investigating the development of novel therapeutic strategies including arsenic trioxide and the mechanisms of RA resistance in myeloid leukaemia.

### ACKNOWLEDGEMENTS

This work was supported by grants from the Ministry of Education, Science and Culture in Japan, the National Grant-in Aid for the Establishment of High-Tech Research Centre in a Private University, and the Keio University Special Grants.

### REFERENCES

Cesano A. Hoxie JA. Lange B. Nowell PC. Bishop J and Santoli D (1992) The severe combined immunodeficient (SCID) mouse as a model for human myeloid leukemias. *Oncogene* **7**: 827–836

Chen G-Q. Zhu J. Shi X-G. Ni J-H. Si G-Y. Jin X-L. Tang W. Li X-S. Xong S-M. Shen Z-X. Sun G-L. Ma J. Zhang T-D. Gazin C. Naoe T. Chen S-J. Wang Z-Y and Chen Z (1996) Use of arsenic trioxide ($As_2O_3$) in the treatment of acute promyelocytic leukemia (APL). I. $As_2O_3$ exerts dose-dependent dual effects on APL cells. *Blood* **88**: 1052–1061

Hiorns LR. Min T. Swansburg GJ. Zelent A. Dyer MJS and Catavsky D (1994) Interstitial insertion of retinoic acid receptor-α gene in acute promyelocytic leukemia with normal chromosomes 15 and 17. *Blood* **83**: 2946–2951

Huang ME. Ye YC. Chen SR. Chai JR. Lu JX. Zhoa L. Gu HT and Wang ZY (1988) Use of all-*trans* retinoic acid in the treatment of acute promyelocytic leukemia. *Blood* **72**: 567–572

Kamada N. Dohy H. Okada K. Oguma N. Kuramoto A. Tanaka K and Uchino H (1981) *In vivo* and *in vitro* activity of neutrophil alkaline phosphatase in acute myelocytic leukemia with 8:21 translocation. *Blood* **58**: 1213–1217

Kanamaru A. Takemoto Y. Tanimoto M. Murakami H. Asou N. Kobayashi T. Kuriyama K. Ohmoto E. Sakamaki H. Tsubaki K. Hiraoka H. Yamada O. Oh H. Saito K. Matsuda S. Minato K. Ueda T and Ohno R (1995) All-*trans* retinoic acid for the treatment of newly diagnosed acute promyelocytic leukemia. *Blood* **85**: 1202–1206

Kitamura T. Tange T. Terashima T. Chiba S. Kuwaki T. Miyagawa K. Piao Y-F. Miyazono K. Urabe A and Takaku F (1989) Establishment and characterization of a unique human cell line that proliferates dependently on GM-CSF. IL-3. or erythropoietin. *J Cell Physiol* **140**: 323–334

Kizaki M. Matsushita H. Takayama N. Muto A. Ueno H. Awaya N. Kawai Y. Asou H. Kamada N and Ikeda Y (1996*a*) Establishment and characterization of a novel acute promyelocytic leukemia cell line (UF-1) with retinoic acid-resistant features. *Blood* **88**: 1824–1833

Kizaki M. Ueno H. Yamazoe Y. Shimada M. Takayama N. Muto A. Matsushita H. Nakajima H. Morikawa M. Koeffler HP and Ikeda Y (1996*b*) Mechanisms of retinoic acid resistance in leukemic cells: possible role of cytochrome P450 and P-glycoprotein. *Blood* **87**: 725–733

Komatsu N. Nakauchi H. Miwa A. Ishihara T. Eguchi M. Moroi M. Okada M. Sato Y. Wada H. Yawata Y. Suda T and Miura Y (1991) Establishment and characterization of a human leukemic cell line with megakaryocytic features: dependency on granulocyte-macrophage colony-stimulating factor. interleukin 3. or erythropoietin for growth and survival. *Cancer Res* **51**: 341–348

Lanotte M. Martin-Thouvenin V. Najiman S. Balerini P. Valensi F and Berger R (1991) NB4. a maturation inducible cell line with t(15:17) marker isolated from a human acute promyelocytic leukemia (M3). *Blood* **77**: 1080–1086

Lapidod T. Sirard C. Vormoor J. Murdoch B. Hoang T. Caceres-Cortes J. Minden M. Peterson B. Caligiuri MA and Dick JE (1994) A cell initiating human acute myeloid leukemia after transplantation into SCID mice. *Nature* **367**: 645–648

Lord CD. Clutterbuck R. Titley J. Ormerod M. Gordon-Smith T. Millar J and Powles R (1991) Growth of primary human acute leukemia in severe combined immunodeficient mice. *Exp Hematol* **19**: 991–993

Miyakawa Y. Fukuchi Y. Ito M. Kobayashi K. Kumamochi T. Ikeda Y. Takeda Y. Tanaka T. Miyasaka M. Nakahata T. Tamaoki N. Nomura T. Ueyama Y and Shimamura K (1996) Establishment of human granulocyte-macrophage colony stimulating factor producing transgenic SCID mice. *Br J Haematol* **95**: 437–442

Namikawa R. Ueda R and Kyoizumi S (1993) Growth of human myeloid leukemias in the human marrow environment of SCID-hu mice. *Blood* **82**: 2526–2536

Sawyers CL. Gishizky ML. Quan S. Golde DW and Witte ON (1992) Propagation of human blastic myeloid leukemias in the SCID mouse. *Blood* **79**: 2089–2098

Takebe Y. Seiki M. Fujisawa J. Hoy P. Yokota K. Arai K. Yoshida M and Arai N (1988) SR alpha promoter: an efficient and versatile mammalian cDNA expression system composed of the simian virus 40 early promoter and the R-U5 segment of human T-cell leukemia virus type I long terminal repeat. *Mol Cell Biol* **8**: 466–472

Terpstra W. Prins A. Visser T. Wognum B. Wagemaker G. Löwenberg B and Wielenga J (1995) Conditions for engraftment of human acute myeloid leukemia (AML) in SCID mice. *Leukemia* **9**: 1573–1577

Uckun FM (1996) Severe combined immunodeficient mouse models of human leukemia. *Blood* **88**: 1135–1146

Warrell Jr RP. Frankel SR. Miller Jr WH. Sheinberg DA. Itri LM. Hittelman WN. Vyas R. Andreeff M. Tafuri A. Jakubowski A. Gabrilove J. Gordon MS and Dmitrovski E (1991) Differentiation therapy of acute promyelocytic leukemia with tretinoin (all-*trans*-retinoic acid). *N Engl J Med* **324**: 1385–1393

Warrell Jr RP. de The H. Wang ZY and Degos L (1993) Acute promyelocytic leukemia. *N Engl J Med* **329**: 177–189

Workman P. Balmain A. Hickman JA. McNally NJ. Mitchison NA. Pierepoint CG. Raymond R. Rowlatt C. Stephens TC and Wallace J (1988) UKCCCR guidelines for the welfare of animals in experimental neoplasia. *Br J Cancer* **58**: 109–113

Yan Y. Salomon O. McGuirk J. Denning D. Fernandez J. Jagiello C. Hai N. Collins N. Steinherz P and O'Reilly RJ (1996) Growth pattern and clinical correlation of subcutaneously inoculated human primary acute leukemias in severe combined immunodeficiency mice. *Blood* **88**: 3137–3147

Zhang S-Y. Zhu J. Chen G-Q. Du X-X. Lu L-J. Zhang Z. Zhong H-J. Chen H-R. Wang Z-Y. Berger R. Lanotte M. Waxman S. Chen Z and Chen S-J (1996) Establishment of a human promyelocytic leukemia-ascites model in SCID mice. *Blood* **87**: 3404–3409

© Cancer Research Campaign 1998


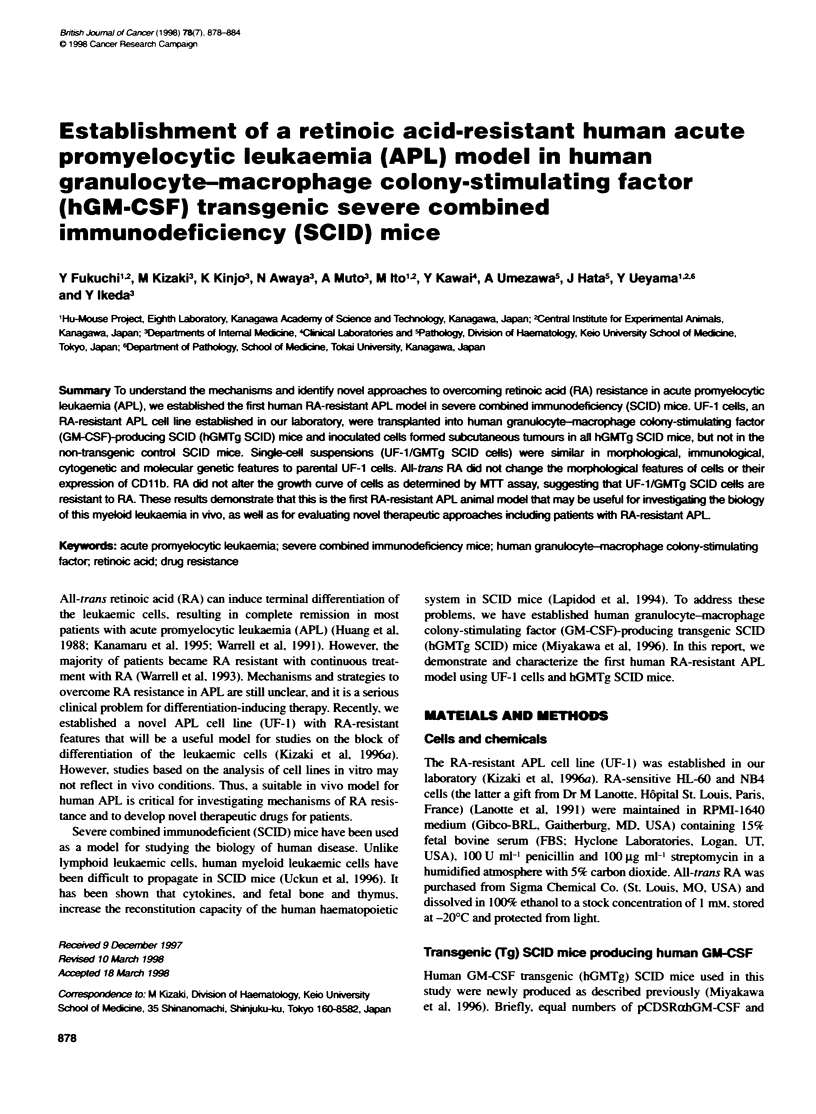

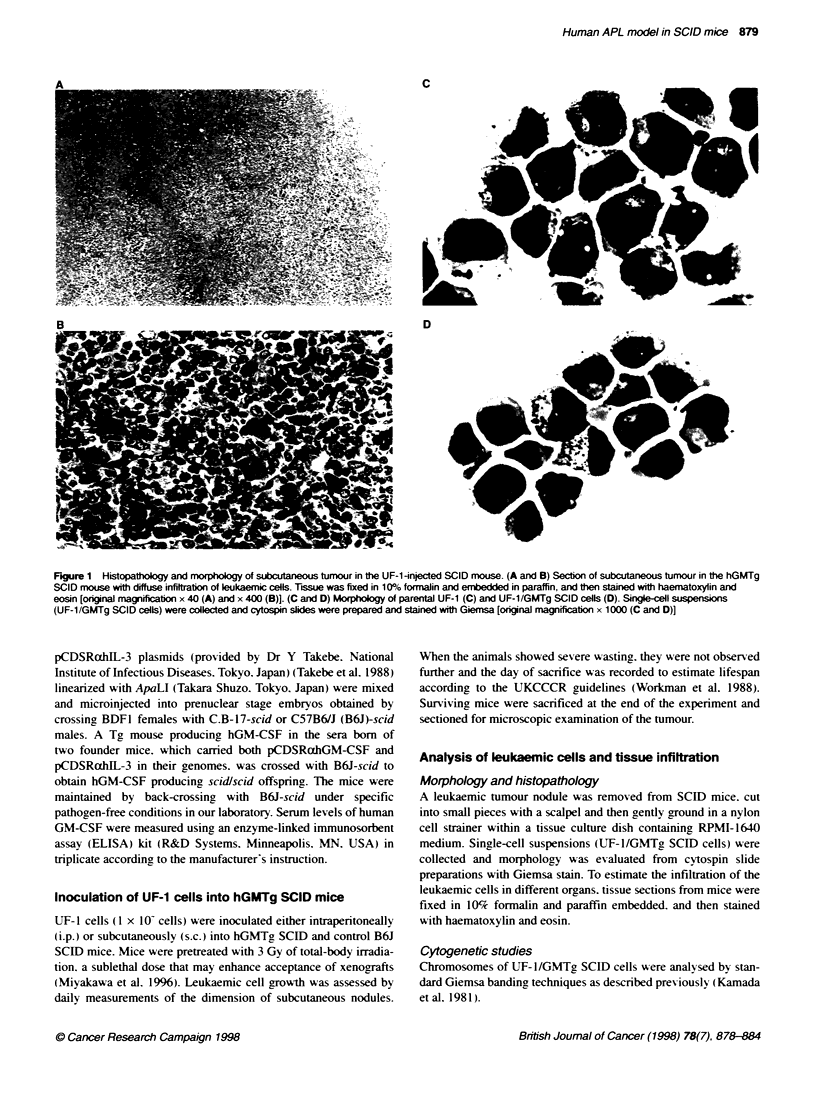

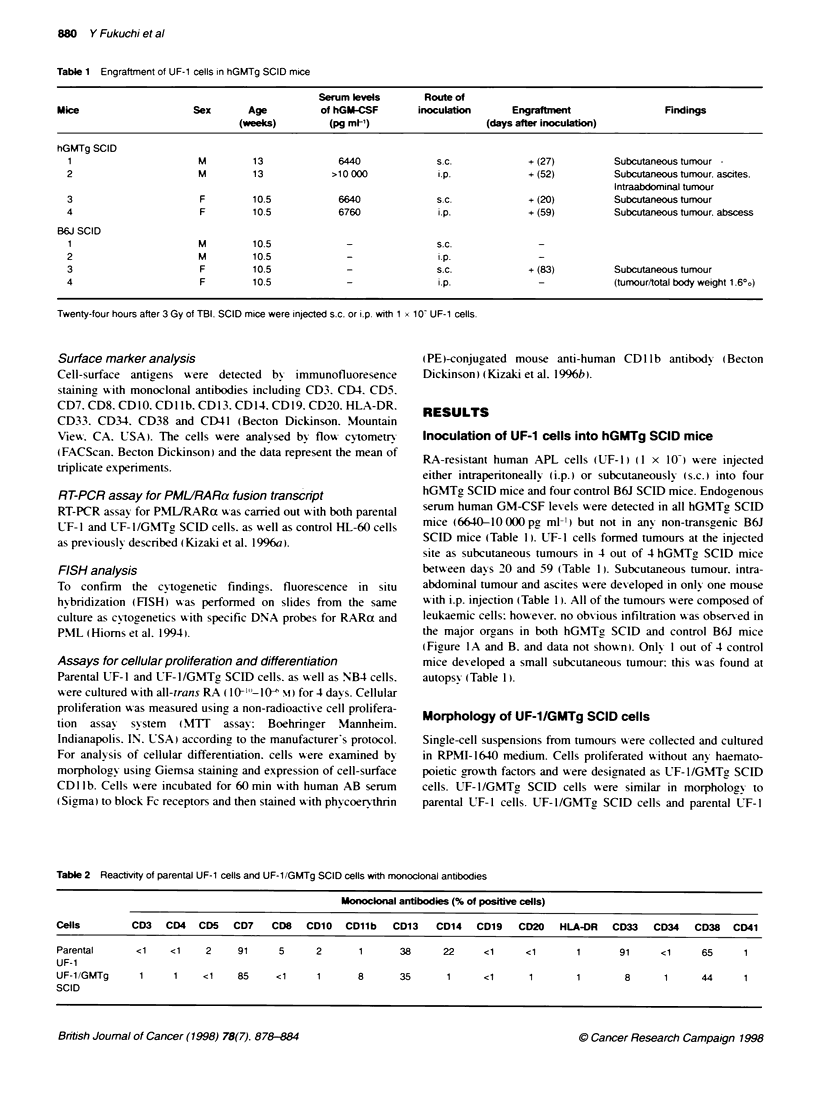

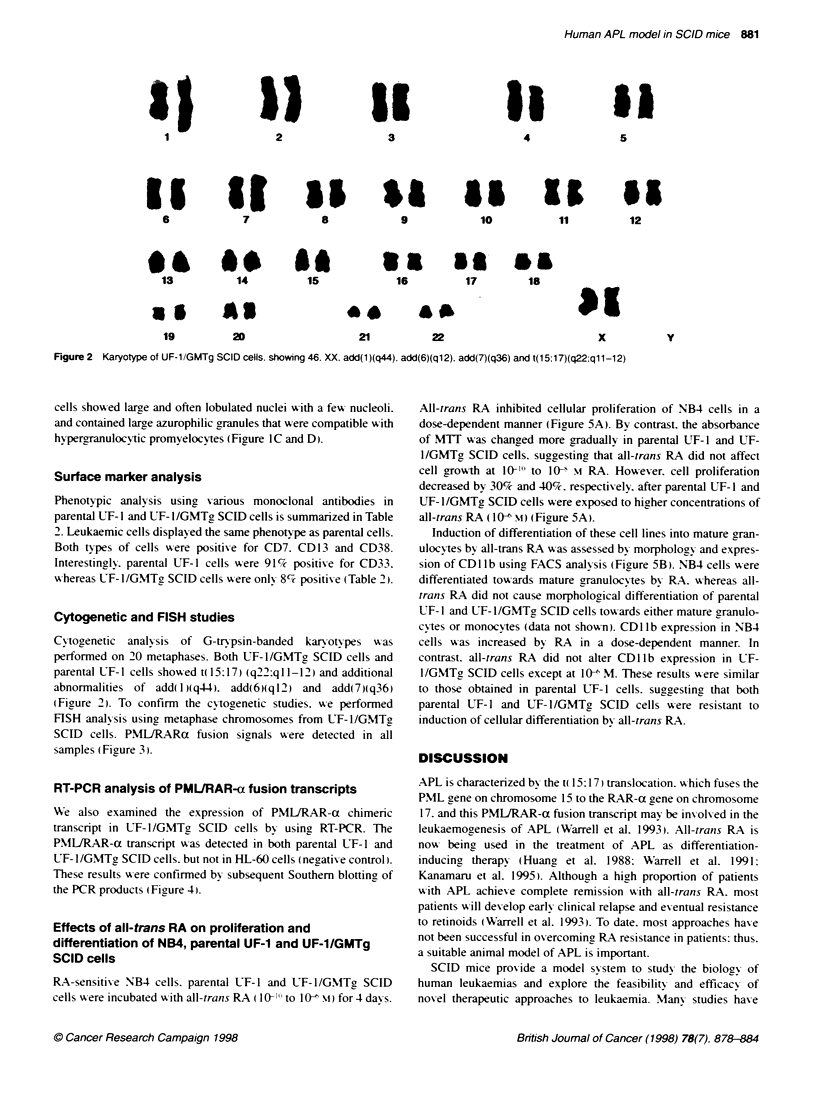

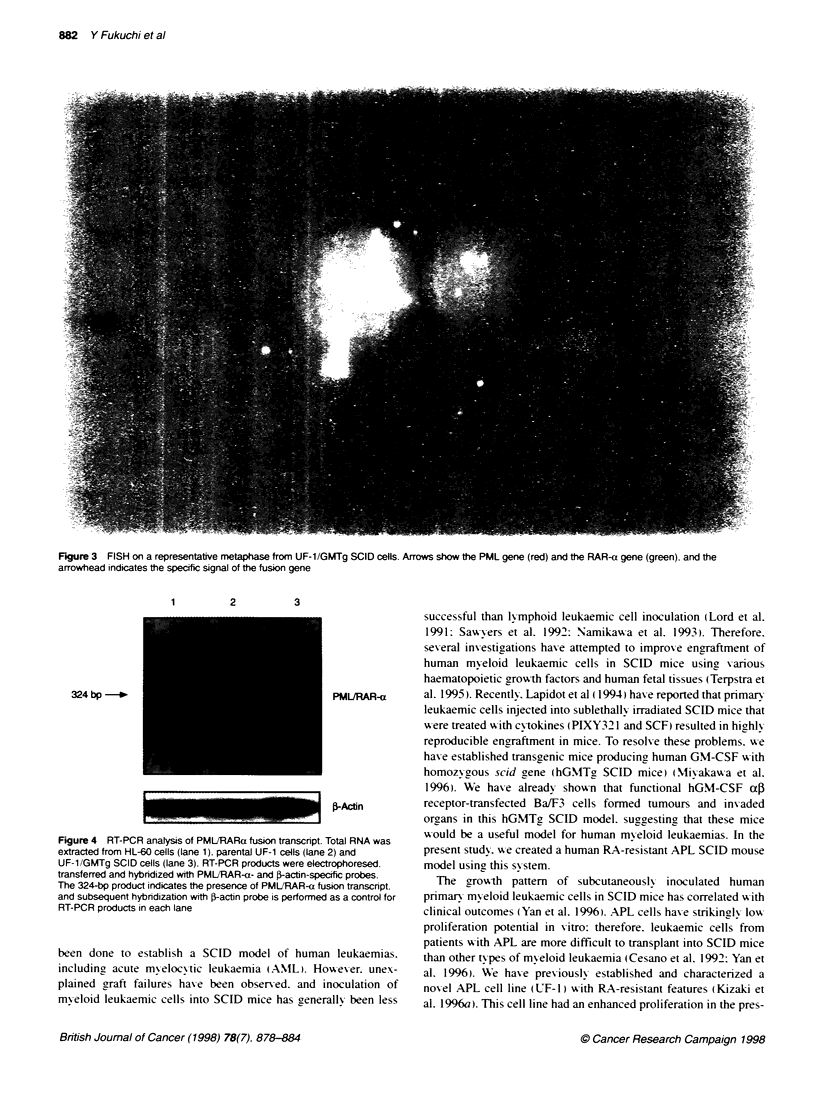

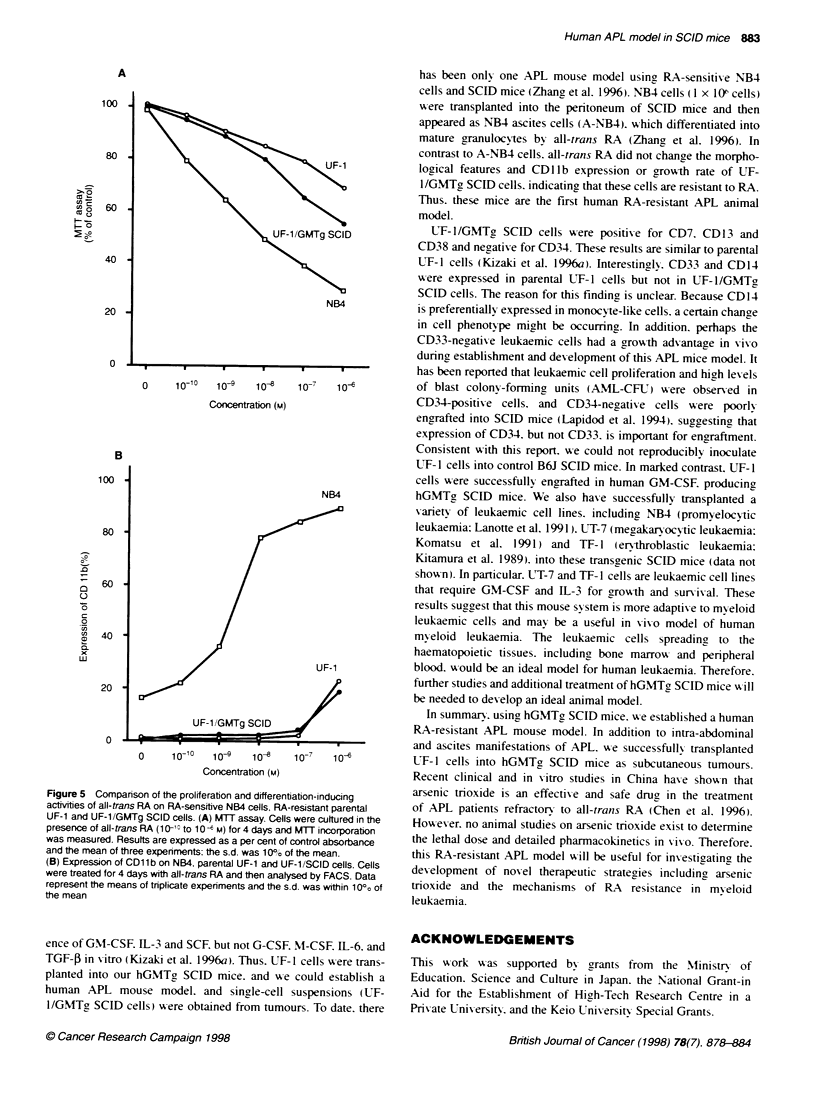

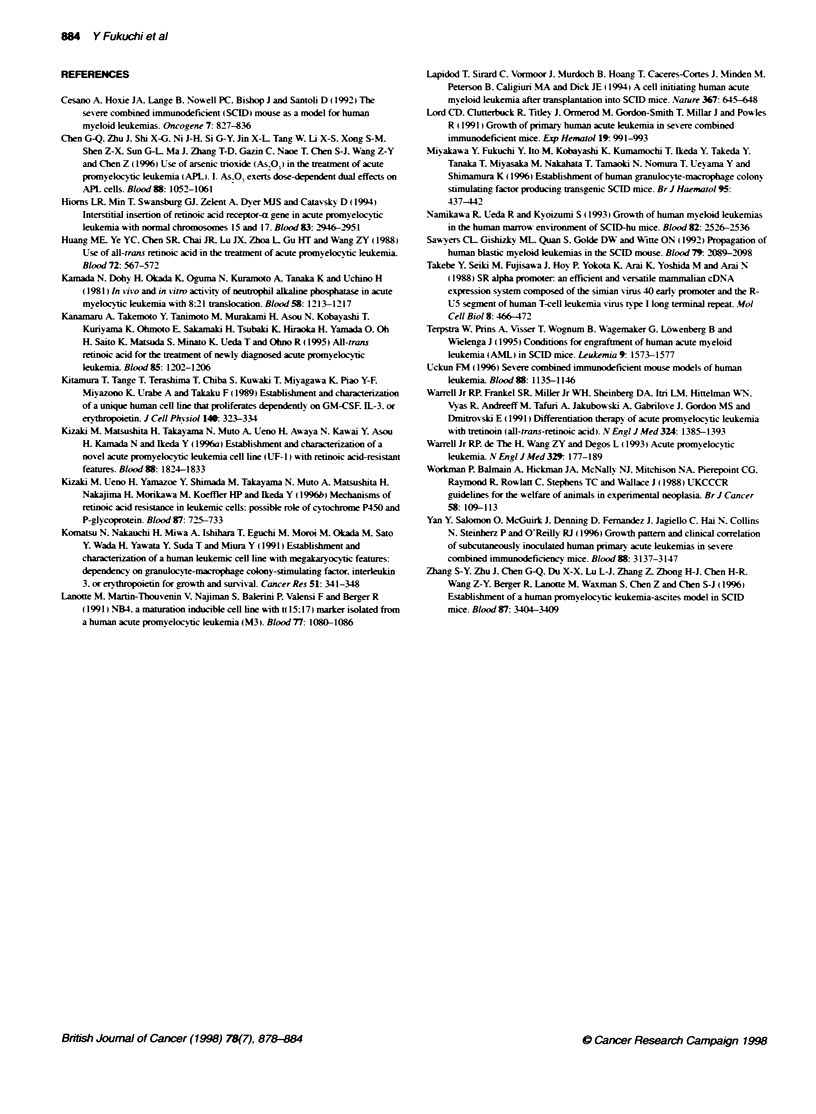


## References

[OCR_00644] Cesano A., Hoxie J. A., Lange B., Nowell P. C., Bishop J., Santoli D. (1992). The severe combined immunodeficient (SCID) mouse as a model for human myeloid leukemias.. Oncogene.

[OCR_00649] Chen G. Q., Zhu J., Shi X. G., Ni J. H., Zhong H. J., Si G. Y., Jin X. L., Tang W., Li X. S., Xong S. M. (1996). In vitro studies on cellular and molecular mechanisms of arsenic trioxide (As2O3) in the treatment of acute promyelocytic leukemia: As2O3 induces NB4 cell apoptosis with downregulation of Bcl-2 expression and modulation of PML-RAR alpha/PML proteins.. Blood.

[OCR_00717] De Lord C., Clutterbuck R., Titley J., Ormerod M., Gordon-Smith T., Millar J., Powles R. (1991). Growth of primary human acute leukemia in severe combined immunodeficient mice.. Exp Hematol.

[OCR_00662] Huang M. E., Ye Y. C., Chen S. R., Chai J. R., Lu J. X., Zhoa L., Gu L. J., Wang Z. Y. (1988). Use of all-trans retinoic acid in the treatment of acute promyelocytic leukemia.. Blood.

[OCR_00667] Kamada N., Dohy H., Okada K., Oguma N., Kuramoto A., Tanaka K., Uchino H. (1981). In vivo and in vitro activity of neutrophil alkaline phosphatase in acute myelocytic leukemia with 8;21 translocation.. Blood.

[OCR_00673] Kanamaru A., Takemoto Y., Tanimoto M., Murakami H., Asou N., Kobayashi T., Kuriyama K., Ohmoto E., Sakamaki H., Tsubaki K. (1995). All-trans retinoic acid for the treatment of newly diagnosed acute promyelocytic leukemia. Japan Adult Leukemia Study Group.. Blood.

[OCR_00680] Kitamura T., Tange T., Terasawa T., Chiba S., Kuwaki T., Miyagawa K., Piao Y. F., Miyazono K., Urabe A., Takaku F. (1989). Establishment and characterization of a unique human cell line that proliferates dependently on GM-CSF, IL-3, or erythropoietin.. J Cell Physiol.

[OCR_00683] Kizaki M., Matsushita H., Takayama N., Muto A., Ueno H., Awaya N., Kawai Y., Asou H., Kamada N., Ikeda Y. (1996). Establishment and characterization of a novel acute promyelocytic leukemia cell line (UF-1) with retinoic acid-resistant features.. Blood.

[OCR_00690] Kizaki M., Ueno H., Yamazoe Y., Shimada M., Takayama N., Muto A., Matsushita H., Nakajima H., Morikawa M., Koeffler H. P. (1996). Mechanisms of retinoid resistance in leukemic cells: possible role of cytochrome P450 and P-glycoprotein.. Blood.

[OCR_00697] Komatsu N., Nakauchi H., Miwa A., Ishihara T., Eguchi M., Moroi M., Okada M., Sato Y., Wada H., Yawata Y. (1991). Establishment and characterization of a human leukemic cell line with megakaryocytic features: dependency on granulocyte-macrophage colony-stimulating factor, interleukin 3, or erythropoietin for growth and survival.. Cancer Res.

[OCR_00706] Lanotte M., Martin-Thouvenin V., Najman S., Balerini P., Valensi F., Berger R. (1991). NB4, a maturation inducible cell line with t(15;17) marker isolated from a human acute promyelocytic leukemia (M3).. Blood.

[OCR_00711] Lapidot T., Sirard C., Vormoor J., Murdoch B., Hoang T., Caceres-Cortes J., Minden M., Paterson B., Caligiuri M. A., Dick J. E. (1994). A cell initiating human acute myeloid leukaemia after transplantation into SCID mice.. Nature.

[OCR_00726] Miyakawa Y., Fukuchi Y., Ito M., Kobayashi K., Kuramochi T., Ikeda Y., Takebe Y., Tanaka T., Miyasaka M., Nakahata T. (1996). Establishment of human granulocyte-macrophage colony stimulating factor producing transgenic SCID mice.. Br J Haematol.

[OCR_00732] Namikawa R., Ueda R., Kyoizumi S. (1993). Growth of human myeloid leukemias in the human marrow environment of SCID-hu mice.. Blood.

[OCR_00734] Sawyers C. L., Gishizky M. L., Quan S., Golde D. W., Witte O. N. (1992). Propagation of human blastic myeloid leukemias in the SCID mouse.. Blood.

[OCR_00737] Takebe Y., Seiki M., Fujisawa J., Hoy P., Yokota K., Arai K., Yoshida M., Arai N. (1988). SR alpha promoter: an efficient and versatile mammalian cDNA expression system composed of the simian virus 40 early promoter and the R-U5 segment of human T-cell leukemia virus type 1 long terminal repeat.. Mol Cell Biol.

[OCR_00747] Terpstra W., Prins A., Visser T., Wognum B., Wagemaker G., Löwenberg B., Wielenga J. (1995). Conditions for engraftment of human acute myeloid leukemia (AML) in SCID mice.. Leukemia.

[OCR_00750] Uckun F. M. (1996). Severe combined immunodeficient mouse models of human leukemia.. Blood.

[OCR_00758] Warrell R. P., Frankel S. R., Miller W. H., Scheinberg D. A., Itri L. M., Hittelman W. N., Vyas R., Andreeff M., Tafuri A., Jakubowski A. (1991). Differentiation therapy of acute promyelocytic leukemia with tretinoin (all-trans-retinoic acid).. N Engl J Med.

[OCR_00763] Warrell R. P., de Thé H., Wang Z. Y., Degos L. (1993). Acute promyelocytic leukemia.. N Engl J Med.

[OCR_00772] Yan Y., Salomon O., McGuirk J., Dennig D., Fernandez J., Jagiello C., Nguyen H., Collins N., Steinherz P., O'Reilly R. J. (1996). Growth pattern and clinical correlation of subcutaneously inoculated human primary acute leukemias in severe combined immunodeficiency mice.. Blood.

[OCR_00778] Zhang S. Y., Zhu J., Chen G. Q., Du X. X., Lu L. J., Zhang Z., Zhong H. J., Chen H. R., Wang Z. Y., Berger R. (1996). Establishment of a human acute promyelocytic leukemia-ascites model in SCID mice.. Blood.

